# Prognostic Significance of the CPS-EG Score in Triple-Negative Breast Cancer Treated with Neoadjuvant Chemotherapy

**DOI:** 10.3390/cancers18142302

**Published:** 2026-07-17

**Authors:** Neslihan Özyurt, Ali Alkan, Burcu Gülbağcı, Mustafa Seyyar, Esra Asik, Mustafa Şahbazlar, Mehmet Türker, Oğuzcan Kınıkoğlu, Tahir Yerlikaya, Gulhan Dinc, Ali Aytaç, Ziya Kalkan, Senar Ebinç, İlkay Gültürk, Merve Keskinkılıç, Zehra Sucuoğlu İşleyen, Dilek Çağlayan, Alper Türkel, Esra Aydın, Teoman Şakalar, Serhat Sekmek, Nilgün Yıldırım, Sinem Akbas, Kerem Okutur, Ahmet Özveren, Bengü Dursun, Orhan Önder Eren, İsmail Beypınar, Pervin Can Şancı, Bahattin Engin Kaya, İlhan Hacıbekiroğlu, Devrim Çabuk, Elanur Karaman, Ömer Acar, Semra Paydaş, Melek Karakurt Eryılmaz, Bilgin Demir, Zeynep Oruç, Mesut Yılmaz, Fatih Selçuk Biricik, Derya Kıvrak Salim, Özgür Tanrıverdi, Mutlu Doğan

**Affiliations:** 1 Department of Medical Oncology, School of Medicine, Ordu University, Ordu 52200, Turkey; 2 Department of Medical Oncology, Faculty of Medicine, Muğla Sıtkı Koçman University, Muğla 48000, Turkey; 3 Department of Medical Oncology, School of Medicine, Sakarya University, Sakarya 54187, Turkey; 4 Department of Medical Oncology, School of Medicine, Kocaeli University, İzmit 41000, Turkey; 5 Department of Medical Oncology, School of Medicine, Karadeniz Technical University, Trabzon 61080, Turkey; 6 Department of Medical Oncology, School of Medicine, Celal Bayar University, Manisa 45140, Turkey; 7 Department of Medical Oncology, School of Medicine, Çukurova University, Adana 01330, Turkey; 8 Department of Medical Oncology, Kartal Training and Research Hospital, İstanbul 34865, Turkey; 9 Department of Medical Oncology, Antalya Training and Research Hospital, Antalya 07100, Turkey; 10 Department of Medical Oncology, Okmeydanı Training and Research Hospital, İstanbul 34098, Turkey; 11 Department of Medical Oncology, School of Medicine, Aydın Adnan Menderes University, Aydın 09100, Turkey; 12 Department of Medical Oncology, School of Medicine, Dicle University, Diyarbakır 21280, Turkey; 13 Department of Medical Oncology, Diyarbakır Training and Research Hospital, Diyarbakır 21070, Turkey; 14 Department of Medical Oncology, Bakırköy Sadi Konuk Training and Research Hospital, İstanbul 34147, Turkey; 15 Department of Medical Oncology, School of Medicine, Dokuz Eylül University, İzmir 35340, Turkey; 16 Department of Medical Oncology, School of Medicine, Bezm-i Alem Vakıf University, İstanbul 34093, Turkey; 17 Department of Medical Oncology, School of Medicine, Necmettin Erbakan University, Konya 42080, Turkey; 18 Department of Medical Oncology, Dr. Abdurrahman Yurtaslan Oncology Training and Research Hospital, Ankara 06200, Turkey; 19 Department of Medical Oncology, School of Medicine, Recep Tayyip Erdoğan University, Rize 53100, Turkey; 20 Department of Medical Oncology, Kahramanmaraş Necip Fazıl City Hospital, Kahramanmaraş 46050, Turkey; 21 Department of Medical Oncology, Ankara Bilkent City Hospital, Ankara 06800, Turkey; 22 Department of Medical Oncology, School of Medicine, Fırat University, Elazığ 23119, Turkey; 23 Department of Medical Oncology, School of Medicine, Koç University, İstanbul 34010, Turkey; 24 Department of Medical Oncology, School of Medicine, İstanbul Arel University, İstanbul 34537, Turkey; 25 Department of Medical Oncology, Acıbadem Kent Hospital, İzmir 35630, Turkey; 26 Department of Medical Oncology, School of Medicine, Ankara University, Ankara 06570, Turkey; 27 Department of Medical Oncology, School of Medicine, Selçuk University, Konya 42130, Turkey; 28 Department of Medical Oncology, School of Medicine, Alanya Alâeddin Keykubat University, Antalya 07425, Turkey

**Keywords:** triple-negative breast cancer, neoadjuvant chemotherapy, CPS-EG score, pathological complete response, disease-free survival, overall survival, prognostic factors, risk stratification

## Abstract

Background/Objectives: Triple-negative breast cancer (TNBC) is an aggressive breast cancer subtype characterized by substantial biological heterogeneity and variable clinical outcomes. Reliable prognostic tools are needed to improve risk stratification following neoadjuvant chemotherapy (NACT). This study aimed to evaluate the association of the Clinical–Pathologic Stage, Estrogen/Grade (CPS-EG) score with pathological complete response (pCR) and survival outcomes in patients with locally advanced TNBC treated with NACT. Methods: In this multicenter retrospective cohort study, 690 patients with locally advanced TNBC treated with NACT between 2010 and 2024 at 25 oncology centers were included. Patients were categorized according to CPS-EG score (≤3 vs. >3). Associations between CPS-EG score, clinicopathological characteristics, pCR, disease-free survival (DFS), and overall survival (OS) were analyzed. Survival outcomes were evaluated using Kaplan–Meier and Cox regression analyses. Results: Patients with a CPS-EG score > 3 had significantly lower pCR rates and higher recurrence rates compared with those with a CPS-EG score ≤ 3 (*p* < 0.001 for both). Five-year OS rates were 83.7% and 54.1% in the CPS-EG ≤ 3 and > 3 groups, respectively, while the corresponding five-year DFS rates were 76.4% and 51.9% (*p* < 0.001 for both). In the multivariable analysis, CPS-EG > 3 remained independently associated with worse OS (HR 1.46, 95% CI 1.08–1.98, *p* = 0.015) and DFS (HR 1.73, 95% CI 1.26–2.38, *p* < 0.001). Conclusions: A higher CPS-EG score is associated with a lower likelihood of achieving pCR and poorer long-term survival outcomes in patients with locally advanced TNBC treated with NACT. The CPS-EG score represents a simple and readily available tool that may support risk stratification and clinical decision-making in routine practice.

## Simple Summary

Triple-negative breast cancer is an aggressive form of breast cancer that often affects younger women and is associated with a higher risk of recurrence and death compared with other breast cancer subtypes. Identifying patients at increased risk of poor outcomes after neoadjuvant chemotherapy remains an important clinical challenge. In this multicenter study, including 690 patients treated at 25 oncology centers, we evaluated the Clinical–Pathologic Stage, Estrogen/Grade (CPS-EG) score as a tool for predicting treatment response and long-term outcomes. Patients with higher CPS-EG scores were less likely to achieve a complete response to treatment and had significantly worse survival outcomes. These findings suggest that the CPS-EG score may help clinicians identify high-risk patients using routinely available clinical and pathological information, supporting more individualized follow-up and treatment strategies in triple-negative breast cancer.

## 1. Introduction

BC remains one of the most frequently diagnosed malignancies and a leading cause of cancer-related mortality among women worldwide, accounting for approximately 1.2–1.4 million new cases and nearly 400,000 deaths annually [1]. TNBC, defined by the absence of estrogen receptor (ER), progesterone receptor (PR), and HER2 expression, constitutes approximately 15–20% of all breast cancers [2] and is characterized by aggressive clinical behavior, high rates of early recurrence, and limited therapeutic options [3]. Despite being traditionally grouped as a single entity, TNBC is biologically heterogeneous and comprises multiple molecular subtypes, including basal-like 1, basal-like 2, mesenchymal-like, and luminal androgen receptor (LAR) subtypes [4,5].

NACT represents the standard treatment approach for patients with locally advanced TNBC, providing tumor downstaging, assessment of treatment sensitivity, and guidance for post-neoadjuvant therapeutic decision-making [6]. Pathological complete response (pCR) following NACT is widely recognized as an important prognostic marker and is generally associated with favorable long-term survival outcomes in TNBC. In selected studies, pCR rates following neoadjuvant treatment have reached up to 68% [7]. However, a substantial proportion of patients fail to achieve pCR and remain at considerable risk of recurrence and death despite standard treatment [8,9]. Moreover, increasing evidence suggests that clinically relevant prognostic heterogeneity persists even among patients with residual disease, highlighting the limitations of relying solely on a binary pCR classification for post-treatment risk assessment [10].

Several prognostic and predictive tools have been investigated in TNBC, including the Ki-67 proliferation index [11], tumor-infiltrating lymphocytes (TILs), residual cancer burden (RCB) [12], and pathological response-based endpoints [13,14]. Although these biomarkers provide valuable prognostic information, some are not routinely implemented in clinical practice or require specialized pathological assessment, limiting their applicability in many treatment settings [15]. In contrast, the CPS-EG score is based on routinely available clinicopathological variables, including clinical stage, pathological stage, ER status, and tumor grade, making it a practical and accessible tool for post-neoadjuvant risk stratification [9,16].

Originally developed in hormone receptor-positive breast cancer, CPS-EG has subsequently been evaluated in TNBC cohorts, including prospective analyses [17,18]. However, its prognostic performance in TNBC remains controversial, particularly in comparison with pathological response and pathological staging systems [19]. Furthermore, most previous studies have primarily relied on clinical trial-based populations, which may not fully reflect the heterogeneity and complexity of routine oncology practice.

Given the biological diversity and aggressive clinical course of TNBC, further evaluation of CPS-EG in real-world TNBC populations may provide additional insight into post-neoadjuvant prognostic stratification. Therefore, the present study aimed to investigate the association between CPS-EG score, pathological response, recurrence patterns, and survival outcomes in a large multicenter real-world cohort of patients with locally advanced TNBC treated with neoadjuvant chemotherapy, with particular emphasis on its prognostic performance among patients without pCR [20].

## 2. Materials and Methods

This multicenter retrospective cohort study included 690 female patients diagnosed with locally advanced triple-negative breast cancer (TNBC) across 25 oncology centers in Turkey between January 2010 and January 2024. Eligible patients were aged ≥ 18 years, had histologically confirmed TNBC defined as estrogen receptor (ER)-negative, progesterone receptor (PR)-negative, and HER2-negative disease (HER2 0–1+ or HER2 2+ with negative fluorescence in situ hybridization [FISH]), and were classified as stage T1–T4a, N0–N3, M0 according to the Union for International Cancer Control (UICC) staging system. All patients received neoadjuvant chemotherapy (NACT) followed by definitive surgical resection.

Patients with metastatic disease at diagnosis, persistent inoperable disease after NACT, previous radiotherapy before surgery, prior or synchronous malignancies, male sex, or insufficient clinicopathological information for outcome analysis were excluded.

Clinical, pathological, treatment, and survival data were retrospectively collected from institutional databases between July 2023 and January 2024. Because of the retrospective design and the use of anonymized clinical data, the requirement for written informed consent was waived by the local ethics committee. The study was conducted in accordance with the Declaration of Helsinki and applicable national ethical regulations.

Demographic, clinical, and pathological variables were extracted from electronic medical records and included age at diagnosis, menopausal status, ECOG performance status, comorbidities, tumor location, histological subtype, clinical and pathological TNM stage, lymphovascular invasion (LVI), perineural invasion (PNI), Ki-67 proliferation index, and BRCA1/2 mutation status. BRCA testing was available only when clinically indicated, as genetic testing was not routinely performed throughout the study period because of evolving clinical indications, local availability, and reimbursement policies.

The mean Ki-67 proliferation index was 54.8 ± 25.6%. Because TNBC is generally characterized by high proliferative activity and no universally accepted Ki-67 threshold exists for this subtype, a cut-off value of 70% was selected to identify tumors with particularly high proliferative potential and to improve the discriminatory capacity within the cohort.

Treatment-related variables included the neoadjuvant chemotherapy regimen, receipt of neoadjuvant immunotherapy, surgical procedure, axillary management strategy, and adjuvant systemic and local therapies. All patients received anthracycline- and/or taxane-based NACT according to institutional protocols and contemporary national guidelines. Platinum-containing regimens were preferentially administered to patients with high-grade tumors or suspected BRCA mutations. Pembrolizumab was incorporated into neoadjuvant treatment in a limited number of patients according to clinical indications and drug availability. Given the multicenter design and the prolonged study period, treatment strategies reflected evolving institutional practice and changes in national treatment recommendations.

Following NACT, patients underwent either breast-conserving surgery or mastectomy according to tumor response, breast characteristics, and patient preference. Axillary management consisted of sentinel lymph node biopsy or axillary lymph node dissection depending on nodal status. Adjuvant treatment decisions, including radiotherapy and systemic therapies such as capecitabine for patients without pCR, were determined according to pathological findings and multidisciplinary team recommendations.

The CPS-EG score was calculated using the publicly available online calculator developed by the MD Anderson Cancer Center according to the original scoring system described by Mittendorf et al. [16]. Because all tumors included in this cohort were estrogen receptor (ER)-negative, the ER component of the score was assigned a value of zero for every patient.

Consequently, no patient had a CPS-EG score of 0, and prognostic discrimination within this cohort was driven by the remaining score components, namely clinical stage, pathological stage, and tumor grade.

Consistent with previous studies evaluating the prognostic utility of CPS-EG after neoadjuvant therapy [21,22,23,24], patients were categorized into two groups according to their CPS-EG score (≤3 vs. >3).

Residual Cancer Burden (RCB) is a validated tool for quantifying residual disease following neoadjuvant therapy. However, RCB could not be calculated because the pathological variables required for standardized RCB assessment—including residual tumor bed dimensions, tumor cellularity, in situ disease percentage, number of positive lymph nodes, and largest nodal metastasis—were not consistently documented across the 25 participating centers during the 14-year study period. Therefore, standardized reconstruction of RCB scores for the entire cohort was not feasible.

Instead, treatment response was assessed using pathological complete response (pCR), defined as the absence of invasive carcinoma in both the breast and axillary lymph nodes following NACT (ypT0/is ypN0). Patients with residual invasive disease in either the breast or axillary lymph nodes, including isolated nodal residual disease (ypT0/is ypN+), were classified as non-pCR.

Recurrence was categorized as locoregional or distant. Locoregional recurrence was defined as recurrence involving the ipsilateral breast, chest wall, or ipsilateral axillary lymph nodes. Time to locoregional recurrence and distant metastasis was calculated from the date of definitive surgery to the date of the corresponding event. Among patients who developed recurrence, recurrence patterns and metastatic sites were further evaluated according to CPS-EG group.

The primary endpoint was disease-free survival (DFS), defined as the interval between definitive surgery and the first occurrence of locoregional recurrence, distant metastasis, or death from any cause. Secondary endpoints included overall survival (OS), defined as the interval from definitive surgery to death from any cause, and pathological complete response (pCR). Additional analyses evaluated recurrence patterns and the association between CPS-EG groups and clinicopathological characteristics, including pathological T stage, pathological N stage, lymphovascular invasion, perineural invasion, HER2-low status, and the Ki-67 proliferation index.

Patients who were alive at the last follow-up were censored on the date of last clinical contact.

### Statistical Analysis

Statistical analyses were performed using IBM SPSS Statistics for Windows, version 31.0 (IBM Corp., Armonk, NY, USA). Continuous variables were summarized as mean ± standard deviation (SD) and median (minimum–maximum), whereas categorical variables were presented as frequencies and percentages. The distribution of continuous variables was assessed using skewness and kurtosis measures together with visual inspection of histograms.

Comparisons between CPS-EG groups were performed using the chi-square test for categorical variables and the independent-samples *t*-test for continuous variables, as appropriate. Bonferroni-adjusted post hoc comparisons were applied when multiple categorical comparisons were required.

Disease-free survival (DFS) and overall survival (OS) were estimated using the Kaplan–Meier method, and survival distributions were compared using the log-rank test. Univariable Cox proportional hazards regression analyses were initially performed to identify variables associated with survival outcomes. Variables with a *p*-value < 0.10 in the univariable analyses, together with variables considered clinically relevant, were subsequently entered into multivariable Cox regression models. Highly correlated variables were not included simultaneously in the primary multivariable models to reduce the risk of multicollinearity. Results were reported as hazard ratios (HRs) with 95% confidence intervals (CIs).

The proportional hazards assumption was assessed graphically using log-minus-log survival plots. The discriminative ability of CPS-EG for OS and DFS was quantified using Harrell’s concordance index (C-index). The association between CPS-EG classification and pathological complete response (pCR) was evaluated using the chi-square test.

Additional analyses were performed to assess treatment-related confounding and the robustness of the prognostic association of CPS-EG. Adjuvant capecitabine use was incorporated into the multivariable Cox regression models, and separate sensitivity analyses were conducted after excluding patients who received adjuvant capecitabine. Multiplicative interaction terms were evaluated between CPS-EG classification and adjuvant capecitabine, anthracycline-based treatment, platinum/taxane-based treatment, neoadjuvant immunotherapy, and HER2-low status. Because only a small proportion of patients received neoadjuvant immunotherapy, the corresponding interaction analyses were considered exploratory and were not formally interpreted.

HER2-stratified Cox regression analyses were also performed to evaluate the prognostic association of CPS-EG separately in HER2-zero and HER2-low tumors. An exploratory component-level analysis was conducted by entering the individual clinical and pathological components of CPS-EG into Cox regression models. Variance inflation factors (VIFs) were calculated to assess multicollinearity among the stage-related variables and other CPS-EG components.

Recurrence patterns were compared between CPS-EG groups using the chi-square test. Among patients who experienced recurrence, the distribution of locoregional versus systemic recurrence and the distribution of metastatic sites were evaluated according to CPS-EG classification.

Missingness was quantified for key clinicopathological variables and is reported in Supplementary Table S1. The primary Cox regression analyses were conducted using a complete-case approach. Multiple imputation was not performed because missingness for several variables reflected non-random clinical testing and documentation practices, particularly for BRCA1/2 status. BRCA-tested and untested patients were additionally compared to explore potential selection bias associated with the availability of genetic testing.

All statistical tests were two-sided, and a *p*-value < 0.05 was considered statistically significant.

## 3. Results

### 3.1. Patient Characteristics

A total of 690 female patients with locally advanced TNBC were included in the study. The median age at diagnosis was 49 years (range: 20–85), with 61.2% of patients aged between 40 and 59 years. Premenopausal and postmenopausal status were nearly equally distributed (50.3% and 49.7%, respectively), and most patients had good performance status (ECOG 0 in 74.1%). Comorbidities were present in 35.4% of patients, most commonly hypertension (23.0%) and diabetes mellitus (11.4%).

The majority of tumors were invasive ductal carcinoma (64.6%), with most patients presenting with clinical T2 (64.8%) and N1 disease (50.1%). Grade III tumors accounted for 64.9% of cases. HER2-0 expression was observed in 68.1% of patients, while HER2-low status was present in 31.9%. The mean Ki-67 index was 54.8% (±25.6). BRCA testing was available for 220 patients, among whom 22.7% were BRCA1-positive and 4.5% were BRCA2-positive (Table 1).

### 3.2. Treatment Characteristics and Pathological Response

Anthracycline-based neoadjuvant chemotherapy was administered to 86.2% of patients, whereas 95.2% received platinum- and taxane-containing regimens. Neoadjuvant immunotherapy was administered to 2.2% of patients. Treatment adherence was high, with 93.4% of patients completing the planned neoadjuvant treatment.

Mastectomy was performed in 58.7% of patients and breast-conserving surgery in 41.3%, while axillary dissection was performed in 53.6% of cases. Pathological complete response (pCR) was achieved in 33.3% of patients, whereas 66.7% had residual invasive disease. Nodal clearance (ypN0) was observed in 64.6% of patients. Adjuvant radiotherapy and capecitabine were administered to 86.4% and 46.1% of patients, respectively. Capecitabine use was more frequent among patients with poorer outcomes, likely reflecting its preferential use in higher-risk patients with residual disease following neoadjuvant treatment (Table 2).

### 3.3. Association Between CPS-EG Score and Clinicopathological Features

**Figure 1:**
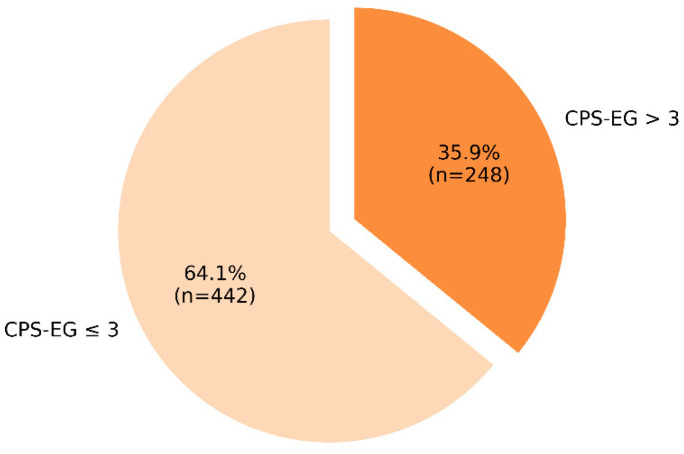
Distribution of CPS-EG classification among patients diagnosed with locally advanced triple-negative breast cancer (TNBC).

The CPS-EG score ranged from 1 to 6 (mean 3.05 ± 1.11). Consistent with previous studies, patients were categorized into two groups according to CPS-EG score (≤3 vs. >3). Among the 690 patients included in the study, 442 (64.1%) were classified as CPS-EG ≤ 3 and 248 (35.9%) as CPS-EG > 3 (Figure 1).

No significant differences were observed between CPS-EG groups regarding anthracycline-based chemotherapy, platinum/taxane-containing regimens, immunotherapy administration, or treatment completion rates (all *p* > 0.05). However, patients with higher CPS-EG scores underwent total mastectomy and axillary dissection significantly more frequently, whereas breast-conserving surgery and sentinel lymph node biopsy were more common among patients with lower CPS-EG scores (both *p* < 0.001) (Table 2).

### 3.4. Survival Outcomes According to CPS-EG Score

During a median follow-up of 6.84 years, 206 patients (29.9%) developed disease recurrence. Among recurrent cases, distant metastases accounted for 73.8% of events, most commonly involving the lungs (24.3%), multiple metastatic sites (22.8%), and bone (17.0%).

Kaplan–Meier analyses demonstrated significantly poorer outcomes among patients with CPS-EG scores > 3. Five-year OS rates were 54.1% and 83.7% in the CPS-EG > 3 and ≤3 groups, respectively (*p* < 0.001). Similarly, five-year DFS rates were 51.9% and 76.4%, respectively (*p* < 0.001).

**Figure 2:**
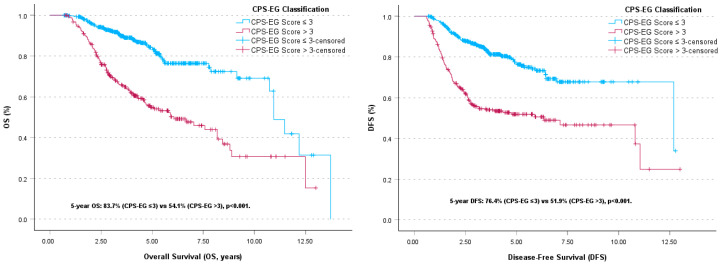
Kaplan–Meier survival curves for overall survival (OS) and disease-free survival (DFS) according to CPS-EG score.

**Figure 3:**
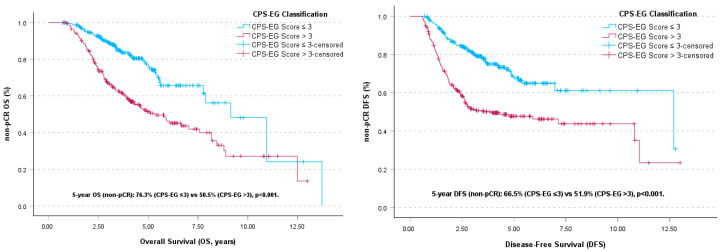
Kaplan–Meier survival curves for overall survival (OS) and disease-free survival (DFS) according to CPS-EG score in the non-pCR patient group.

In the non-pCR subgroup, patients with CPS-EG scores > 3 also experienced significantly worse outcomes, with lower 5-year OS (50.5% vs. 76.3%, *p* < 0.001) and DFS rates (51.9% vs. 66.5%, *p* < 0.001) than those with CPS-EG scores ≤ 3 (Table 3; Figure 2 and Figure 3).

### 3.5. Multivariable Analysis

In multivariable Cox regression analyses, a CPS-EG score > 3 remained independently associated with both inferior OS and DFS after adjustment for clinicopathological and treatment-related variables. Patients with CPS-EG scores > 3 had a significantly increased risk of overall mortality (HR 1.46, 95% CI 1.08–1.98, *p* = 0.015) and recurrence or death (HR 1.73, 95% CI 1.26–2.38, *p* < 0.001) compared with those with CPS-EG scores ≤ 3.

Non-pCR status and residual nodal disease following neoadjuvant therapy (ypN+) also emerged as strong independent adverse prognostic factors in both survival models. In the DFS model, lymphovascular invasion was additionally associated with poorer outcomes, whereas HER2-low status and ECOG performance status ≥ 1 were independently associated with worse OS (Table 4 and Table 5).

### 3.6. Missing Data and BRCA Testing

Missing data were limited for most clinicopathological variables but were more substantial for selected biomarkers. Ki-67 category was missing in 28 patients (4.1%), lymphovascular invasion (LVI) in 58 (8.4%), perineural invasion (PNI) in 78 (11.3%), and histological subtype in 42 (6.1%). BRCA status was available for 220 patients (31.9%) and unavailable for 470 patients (68.1%). Patients who underwent BRCA testing differed from untested patients with respect to age, menopausal status, comorbidities, clinical T stage, clinical N stage, and HER2-low status, indicating that BRCA testing was not performed randomly across the cohort. Therefore, BRCA status was not included in the primary multivariable Cox regression models. The proportions of missing data are summarized in Supplementary Table S1.

### 3.7. Sensitivity Analyses

To evaluate the robustness of the prognostic effect of CPS-EG, additional sensitivity analyses were performed. After adjustment for adjuvant capecitabine use, CPS-EG > 3 remained independently associated with inferior overall survival (HR 1.64, 95% CI 1.15–2.34; *p* = 0.006) and disease-free survival (HR 1.72, 95% CI 1.25–2.36; *p* = 0.001). Adjuvant capecitabine itself was not independently associated with OS (HR 1.12, 95% CI 0.80–1.58; *p* = 0.506) or DFS (HR 1.13, 95% CI 0.82–1.56; *p* = 0.438).

A second sensitivity analysis excluding patients who received adjuvant capecitabine demonstrated that CPS-EG > 3 remained significantly associated with inferior DFS (HR 2.17, 95% CI 1.30–3.61; *p* = 0.003) and showed a similar effect size for OS (HR 1.67, 95% CI 0.95–2.92; *p* = 0.072), although statistical significance was not reached.

### 3.8. Interaction Analyses

No statistically significant interaction was observed between CPS-EG classification and adjuvant capecitabine, anthracycline-based treatment, platinum/taxane-based treatment, or HER2-low status for either OS or DFS.

Specifically, the CPS-EG × HER2-low interaction term was not statistically significant for OS (*p* = 0.885) or DFS (*p* = 0.709), indicating no evidence that the prognostic effect of CPS-EG differed according to HER2 status.

In HER2-zero tumors, CPS-EG > 3 was independently associated with inferior OS (HR 1.73, *p* = 0.017) and DFS (HR 1.71, *p* = 0.015). In HER2-low tumors, the association was similar in magnitude, reaching statistical significance for DFS (HR 1.81, *p* = 0.018) and showing a borderline association for OS (HR 1.77, *p* = 0.056). These findings suggest a consistent prognostic effect of CPS-EG across HER2 subgroups (Supplementary Table S2).

### 3.9. Sensitivity Analysis According to the Definition of Pathological Complete Response

Because the definition of pathological complete response (pCR) may influence prognostic analyses, an additional sensitivity analysis was performed using the strict definition of pCR (ypT0/is ypN0). Thirty-two patients had isolated residual nodal disease (ypT0/is ypN+) and were therefore classified as non-pCR in this analysis.

Using the strict definition, 230 patients (33.3%) achieved pCR. Repeating the multivariable Cox regression analyses demonstrated that CPS-EG > 3 remained independently associated with inferior OS (HR 1.64, 95% CI 1.15–2.35; *p* = 0.006) and DFS (HR 1.71, 95% CI 1.24–2.36; *p* = 0.001), confirming the robustness of the prognostic effect irrespective of the pCR definition used.

### 3.10. Exploratory Component Analyses

To further explore the contribution of the individual CPS-EG components, an exploratory Cox regression analysis was performed using the clinical and pathological staging variables separately. Clinical T stage, clinical N stage, pathological T stage, and pathological N stage contributed significantly to survival outcomes, whereas tumor grade did not retain independent prognostic significance after adjustment.

Variance inflation factor (VIF) analysis demonstrated expected correlations among the stage-related variables (clinical T: 8.72, clinical N: 6.06, pathological T: 7.37), reflecting the overlap among the components incorporated into the CPS-EG score. These findings support the use of the composite CPS-EG score as the primary prognostic variable while highlighting the inherent correlation among its individual components (Supplementary Table S3).

### 3.11. Recurrence Pattern Analyses

Among the 206 patients who developed disease recurrence, 152 (73.8%) experienced systemic recurrence and 54 (26.2%) developed isolated locoregional recurrence. The proportion of systemic versus locoregional recurrence did not differ significantly according to CPS-EG group (*p* = 0.772). Likewise, the distribution of specific metastatic sites was comparable between CPS-EG groups (*p* = 0.609).

These findings indicate that, although CPS-EG was associated with the overall risk of recurrence, it did not identify a distinct pattern of metastatic spread in this cohort.

## 4. Discussion

The present multicenter study represents one of the largest real-world evaluations of the Clinical–Pathologic Stage–Estrogen/Grade (CPS-EG) scoring system specifically in patients with locally advanced triple-negative breast cancer (TNBC) treated with neoadjuvant chemotherapy. Although CPS-EG was originally developed in a broader breast cancer population, our findings demonstrate that the score retained independent prognostic value in an exclusively ER-negative cohort. Patients with CPS-EG scores > 3 experienced significantly inferior pathological complete response (pCR), disease-free survival (DFS), and overall survival (OS), and these associations remained robust after multivariable adjustment and multiple sensitivity analyses. Nevertheless, the observed discriminatory performance was moderate, indicating that CPS-EG should be regarded as a complementary clinicopathological prognostic tool rather than a stand-alone prediction model.

Pathological complete response remains one of the strongest surrogate markers of favorable long-term outcome following neoadjuvant chemotherapy in TNBC. The landmark pooled CTNeoBC analysis demonstrated that achievement of pCR is strongly associated with improved event-free and overall survival, particularly in aggressive breast cancer subtypes such as TNBC [8]. More recently, comprehensive meta-analyses by Spring et al. [19], Yoshino et al. [25], and Conforti et al. [26] further confirmed that although pCR is an important surrogate endpoint at the trial level, substantial prognostic heterogeneity persists among individual patients who fail to achieve pCR. Accordingly, binary classification according to pCR alone may not adequately reflect the biological diversity of residual disease. Consistent with these observations, our study demonstrated that patients with CPS-EG > 3 had significantly poorer DFS and OS even within the non-pCR subgroup, suggesting that CPS-EG captures clinically relevant prognostic information beyond pathological response alone.

The biological rationale underlying CPS-EG also supports its applicability in TNBC. Unlike pCR, which reflects only residual disease after treatment, CPS-EG integrates both pretreatment and posttreatment clinicopathological information, including baseline clinical stage, pathological stage, tumor grade, and estrogen receptor status [9,16]. Although the ER component was uniformly zero in the present cohort, the remaining components continued to provide meaningful prognostic stratification. This finding suggests that the prognostic value of CPS-EG in TNBC is primarily driven by disease burden before treatment and the extent of residual disease after neoadjuvant therapy. At the same time, the lack of variability in ER status likely contributes to the moderate discrimination observed in our study and highlights an important limitation of applying a score originally developed for mixed breast cancer populations to a biologically distinct TNBC cohort. Future TNBC-specific prognostic models incorporating disease-specific biomarkers may therefore further improve risk stratification.

Several previous studies have evaluated CPS-EG in patients receiving neoadjuvant treatment. Mittendorf et al. originally demonstrated that the incorporation of pathological stage, tumor grade, and ER status substantially improved prognostic discrimination compared with clinical staging alone [16]. Subsequently, Michel et al. confirmed the clinical utility of CPS-EG for locoregional risk stratification following neoadjuvant chemotherapy [27,28,29], whereas Xu et al. validated both CPS-EG and Neo-Bioscore in an independent Asian cohort [20]. More recently, Marmé et al. reported that CPS-EG effectively identified prognostic heterogeneity among patients with TNBC who failed to achieve pCR [18], findings that were independently confirmed in a contemporary Turkish cohort by Öner et al. [30]. Our results are highly consistent with these observations and extend the existing evidence by demonstrating the prognostic relevance of CPS-EG in one of the largest multicenter real-world TNBC cohorts reported to date.

The contemporary clinical relevance of CPS-EG is further supported by its incorporation into several major post-neoadjuvant clinical trials. In the OlympiA trial, CPS-EG contributed to identifying patients with germline BRCA1/2 mutations who derived benefit from adjuvant olaparib [22,31]. Likewise, the PENELOPE-B trial used CPS-EG together with residual disease burden to define a population at particularly high risk of recurrence following neoadjuvant chemotherapy [23].

Similarly, the ongoing SASCIA trial employs CPS-EG-based eligibility criteria to evaluate sacituzumab govitecan in patients with residual HER2-negative breast cancer after standard neoadjuvant treatment [24]. Collectively, these studies indicate that CPS-EG has evolved beyond a purely prognostic score and is increasingly being incorporated into risk-adapted post-neoadjuvant treatment strategies. Our findings provide additional real-world support for this approach, although the moderate discriminatory performance observed in our cohort suggests that CPS-EG alone is unlikely to be sufficient for individualized treatment decisions.

An important strength of the present study is the robustness of the observed prognostic associations across multiple sensitivity analyses. Adjustment for adjuvant capecitabine did not materially alter the relationship between CPS-EG and survival, and exclusion of capecitabine-treated patients yielded similar effect estimates despite reduced statistical power. Likewise, no statistically significant interaction was observed between CPS-EG and platinum-containing chemotherapy, anthracycline-based treatment, adjuvant capecitabine, or HER2-low status. These findings suggest that the prognostic value of CPS-EG is relatively stable across contemporary treatment approaches and is unlikely to be explained solely by treatment allocation or post-neoadjuvant therapeutic intensification.

Another noteworthy finding of the present study concerns the potential influence of HER2-low expression on prognosis. Although HER2-low tumors were more frequently observed among patients with higher CPS-EG scores and HER2-low status was independently associated with inferior OS in the primary multivariable model, interaction analyses demonstrated no statistically significant modification of the prognostic effect of CPS-EG according to HER2 status. Furthermore, subgroup analyses revealed a similar direction and magnitude of association between CPS-EG and survival in both HER2-zero and HER2-low tumors. These findings suggest that the prognostic performance of CPS-EG is broadly preserved irrespective of HER2-low status. Recent studies have produced conflicting results regarding the prognostic significance of HER2-low disease in TNBC. While Shi et al. [32,33] and Lee et al. [34] reported differences in treatment response and survival between HER2-low and HER2-zero tumors, Roussot et al. demonstrated that CPS-EG retained prognostic value across both HER2 subgroups [32]. Likewise, Fusco and Viale emphasized that HER2-low should currently be regarded primarily as a therapeutic rather than a distinct biological entity, although its molecular heterogeneity remains under active investigation [35]. Together with our previous multicenter TOG analysis evaluating HER2 status in early TNBC [36], the present findings support the concept that HER2-low expression contributes to the biological diversity within TNBC but does not appear to substantially modify the prognostic utility of CPS-EG. Detailed results of the HER2-stratified analyses are provided in Supplementary Table S4.

An important issue raised by the reviewers was the absence of Residual Cancer Burden (RCB) assessment. RCB is one of the most extensively validated prognostic systems for patients with residual disease following neoadjuvant chemotherapy and has consistently demonstrated excellent prognostic discrimination across breast cancer subtypes, particularly in TNBC [13]. Choi et al. further reported that although CPS-EG remained prognostic, RCB provided superior discrimination in patients receiving neoadjuvant treatment [37]. Unfortunately, reliable retrospective calculation of RCB was not possible in the present study because the standardized pathological variables required for RCB scoring—including residual tumor bed dimensions, overall tumor cellularity, percentage of in situ disease, and detailed nodal tumor burden—were not uniformly documented across the 25 participating centers during the 14-year study period. Consequently, our study cannot determine whether CPS-EG provides incremental prognostic information beyond RCB. Rather than representing a substitute for RCB, CPS-EG should be viewed as a practical complementary clinicopathological tool that may be particularly useful in routine clinical settings where standardized RCB assessment is unavailable or was not historically performed.

The interpretation of our findings should also consider the evolving treatment landscape of TNBC during the study period [38]. Between 2010 and 2024, systemic treatment strategies changed substantially, including the increasing use of platinum-containing neoadjuvant regimens and the introduction of pembrolizumab-based neoadjuvant immunotherapy following the KEYNOTE-522 trial [39]. Similarly, post-neoadjuvant treatment options expanded with the incorporation of capecitabine, PARP inhibitors for germline BRCA-mutated disease, and antibody–drug conjugates for HER2-low breast cancer [22,24,31,35]. Although platinum-containing chemotherapy was widely administered in our cohort, only a small proportion of patients received pembrolizumab, precluding meaningful analyses within the contemporary immunotherapy era. Therefore, our findings should primarily be interpreted within the context of conventional neoadjuvant chemotherapy, and additional validation in modern immunotherapy-treated cohorts remains warranted.

Several strengths of the present study deserve emphasis. First, this represents one of the largest multicenter real-world analyses evaluating CPS-EG specifically in locally advanced TNBC. The inclusion of 690 patients from 25 oncology centers enhances the generalizability of the findings and reflects routine clinical practice more accurately than highly selected clinical trial populations. Second, comprehensive clinicopathological and survival data enabled detailed subgroup analyses, including evaluation within the non-pCR population, treatment-adjusted sensitivity analyses, interaction analyses, and assessment of recurrence patterns. Third, the consistency of the prognostic effect across multiple analyses supports the robustness of the observed associations despite the inherent limitations of retrospective observational research.

Nevertheless, several limitations should be acknowledged. The retrospective design inevitably introduces the possibility of selection bias, residual confounding, and interinstitutional heterogeneity in pathological assessment and treatment decisions. Although participating centers followed contemporary national and international treatment recommendations, management strategies evolved considerably throughout the study period. In particular, the increasing use of platinum-based chemotherapy and the recent introduction of immunotherapy may have influenced outcomes. Because the year of diagnosis was unavailable in the anonymized analytical dataset, formal treatment-era analyses could not be performed. Likewise, BRCA testing was available in only 31.9% of patients and was clearly non-random, reflecting evolving testing indications and reimbursement policies. Consequently, residual confounding related to inherited cancer susceptibility cannot be completely excluded.

Another important limitation is that CPS-EG was originally developed in breast cancer populations that included hormone receptor-positive disease. Because all tumors in the present study were ER-negative, the ER component did not contribute to score variability, and no patient had a CPS-EG score of zero. Furthermore, translational biomarkers with established prognostic relevance in contemporary TNBC—including tumor-infiltrating lymphocytes (TILs), androgen receptor expression, homologous recombination deficiency (HRD), circulating tumor DNA (ctDNA), and genomic signatures—were unavailable in this retrospective dataset. Therefore, the present study should be regarded as a validation of a readily applicable clinicopathological score rather than a comprehensive biological prognostic model.

Future investigations should focus on prospective external validation of CPS-EG in contemporary TNBC cohorts receiving pembrolizumab-containing neoadjuvant regimens. Direct comparison with Residual Cancer Burden, integration with emerging biomarkers such as TIL density, ctDNA, BRCA/HRD status, androgen receptor expression, and transcriptomic classifiers, together with evaluation using decision-curve analysis and machine learning-assisted prediction models, may further improve individualized post-neoadjuvant risk stratification. Such multidimensional approaches may ultimately facilitate more precise treatment escalation and de-escalation strategies for patients with residual TNBC.

## 5. Conclusions

In conclusion, CPS-EG was independently associated with pathological complete response, recurrence risk, disease-free survival, and overall survival in patients with locally advanced TNBC treated with neoadjuvant chemotherapy. However, its discriminatory performance was moderate, and the score should not be interpreted as a stand-alone prognostic model. Instead, CPS-EG may provide complementary prognostic information using routinely available clinicopathological variables, particularly in settings where standardized Residual Cancer Burden (RCB) assessment is unavailable. Prospective external validation in independent contemporary TNBC cohorts, especially those receiving immunotherapy-containing neoadjuvant regimens, is required before broader clinical implementation can be recommended.

## 6. Strengths, Limitations, and Future Directions

This study has several important strengths. To our knowledge, it represents one of the largest multicenter real-world cohort studies evaluating the prognostic performance of the CPS-EG score specifically in patients with locally advanced TNBC treated with neoadjuvant chemotherapy. The inclusion of 690 patients from 25 oncology centers enhances the generalizability of the findings and reflects routine clinical practice beyond highly selected clinical trial populations. Furthermore, the large sample size enabled comprehensive subgroup, sensitivity, and interaction analyses, including evaluation according to pathological response, treatment-related variables, HER2-low status, and recurrence patterns. The consistency of the findings across these analyses supports the robustness of the observed prognostic associations.

Several limitations should also be acknowledged. First, the retrospective multicenter design inevitably introduces the possibility of selection bias, reporting bias, residual confounding, and interinstitutional variability in pathological assessment and treatment decisions. Although participating centers generally followed contemporary treatment guidelines, management strategies evolved substantially during the 2010–2024 study period, particularly regarding the increasing use of platinum-containing chemotherapy and the introduction of pembrolizumab-based neoadjuvant immunotherapy. Because the calendar year was unavailable in the anonymized analytical dataset, formal treatment-era analyses could not be performed. Moreover, only 15 patients received immunotherapy, precluding meaningful evaluation of outcomes in the contemporary immunotherapy era.

Second, BRCA testing was available for only 31.9% of patients and was performed non-randomly according to evolving clinical indications and reimbursement policies. Consequently, inherited genetic susceptibility could not be comprehensively incorporated into the prognostic analyses. Similarly, complete-case analysis was adopted because missingness was substantial and clearly non-random for several variables, particularly BRCA status.

Another important limitation is the absence of Residual Cancer Burden (RCB) assessment. Although RCB is one of the most extensively validated prognostic tools following neoadjuvant therapy, reliable retrospective reconstruction was not possible because the pathological variables required for standardized RCB calculation—including residual tumor bed dimensions, tumor cellularity, in situ disease, and detailed nodal tumor burden—were not uniformly recorded across participating centers throughout the study period. Consequently, the present study cannot determine whether CPS-EG provides incremental prognostic value beyond RCB and should therefore be interpreted as evaluating a complementary rather than competing prognostic approach.

Although HER2-low tumors were associated with adverse clinicopathological characteristics, interaction analyses demonstrated no statistically significant modification of the prognostic effect of CPS-EG according to HER2 status [40].

Likewise, important contemporary biomarkers, including tumor-infiltrating lymphocytes (TILs), androgen receptor (AR) expression, homologous recombination deficiency (HRD), circulating tumor DNA (ctDNA), and transcriptomic signatures, were unavailable because of the retrospective design. Therefore, CPS-EG should be regarded as a practical clinicopathological prognostic tool rather than a comprehensive biological prediction model.

Future prospective studies should externally validate CPS-EG in independent TNBC cohorts treated with contemporary immunotherapy-containing neoadjuvant regimens. Direct comparison with standardized RCB assessment and integration with emerging biomarkers—including TIL density, ctDNA, BRCA/HRD status, AR expression, and transcriptomic classifiers—may further improve individualized post-neoadjuvant risk stratification. In addition, decision-curve analysis and modern prediction approaches incorporating digital pathology and artificial intelligence may help determine the incremental clinical value of CPS-EG within future precision oncology strategies.

## Figures and Tables

**Table 1:** Demographic, Clinical, and Tumor Characteristics of Patients with Locally Advanced Triple-Negative Breast Cancer (TNBC) Stratified by CPS-EG Classification.

Variables		Total		CPS-EG Classification				*p*
				CPS-EG Score ≤ 3 (*n* = 442)		CPS-EG Score > 3 (*n* = 248)		
		*n*	%	*n*	%	*n*	%	
Age at diagnosis	<50 years	365	52.9	232	52.5	133	53.6	0.835
	≥50 years	325	47.1	210	47.5	115	46.4	
Menopausal status	Premenopausal	347	50.3	216	48.9	131	52.8	0.359
	Postmenopausal	343	49.7	226	51.1	117	47.2	
ECOG status	ECOG 0	511	74.1	326	73.8	185	74.6	0.880
	ECOG ≥ 1	179	25.9	116	26.2	63	25.4	
Comorbidity	No	446	64.6	284	64.3	162	65.3	0.842
	Yes	244	35.4	158	35.7	86	34.7	
HT	No	531	77.0	342	77.4	189	76.2	0.799
	Yes	159	23.0	100	22.6	59	23.8	
DM	No	611	88.6	391	88.5	220	88.7	1.000
	Yes	79	11.4	51	11.5	28	11.3	
COPD	No	679	98.4	436	98.6	243	98.0	0.729
	Yes	11	1.6	6	1.4	5	2.0	
Cardiovascular disease (CAD/CHF)	No	649	94.1	414	93.7	235	94.8	0.678
	Yes	41	5.9	28	6.3	13	5.2	
Tumor location	Lateral	417	60.4	271	61.3	146	58.9	0.781
	Medial	160	23.2	99	22.4	61	24.6	
	Other	113	16.4	72	16.3	41	16.5	
Histologic subtype	IDC	446	64.6	297	67.2	149	60.1	0.100
	Non-IDC	202	29.3	123	27.8	79	31.9	
	Unknown histology	42	6.1	22	5.0	20	8.1	
Clinical T stage	T1–T2	513	74.3	368	83.3	145	58.5	<0.001 *
	T3–T4	177	25.7	74	16.7	103	41.5	
Clinical N stage	N0	123	17.8	111	25.1	12	4.8	<0.001 *
	N+	567	82.2	331	74.9	236	95.2	
HER2 status	HER2-zero	470	68.1	318	71.9	152	61.3	0.005 *
	HER2-low	220	31.9	124	28.1	96	38.7	
LVI	Absent	435	68.8	299	74.8	136	58.6	<0.001 *
	Present	197	31.2	101	25.3	96	41.4	
PNI	Absent	535	87.4	347	88.7	188	85.1	0.234
	Present	77	12.6	44	11.3	33	14.9	
Tumor grade	I	12	1.7	10	2.3	2	0.8	<0.001 *
	II	230	33.3	203	45.9	27	10.9	
	III	448	64.9	229	51.8	219	88.3	
Ki-67 proliferation index (%)	<70	394	59.5	268	62.6	126	53.8	0.034 *
	≥70	268	40.5	160	37.4	108	46.2	
Variable		Mean ± SD Median (Min.–Max.)		Mean ± SD Median (Min.–Max.)		Mean ± SD Median (Min.–Max.)		*p*
Age at diagnosis		49.53 ± 11.57 49 (20–85)		49.55 ± 11.13 49 (20–84)		49.50 ± 12.33 48 (23–85)		0.957

Abbreviations: HT: hypertension; DM: diabetes mellitus; COPD: chronic obstructive pulmonary disease; CAD: coronary artery disease; CHF: congestive heart failure; IDC: invasive ductal carcinoma; LVI: lymphovascular invasion; PNI: perineural invasion; CPS-EG: Clinical–Pathologic Stage–Estrogen/Grade. Mean: Mean; SD: Standard deviation; Med.: Median; Min.: Minimum; Max.: Maximum. The Chi-square (χ^2^) test was used for comparisons of categorical variables, while the independent-samples *t*-test was used for comparisons of continuous variables. Statistical significance was accepted as * *p* < 0.05.

**Table 2:** Treatment Characteristics and Pathological Response According to CPS-EG Classification in Patients with Locally Advanced TNBC.

Variables		Total		CPS-EG Score ≤ 3 (*n* = 442)				*p*
				CPS-EG Score ≤ 3 (*n* = 442)		CPS-EG Score > 3 (*n* = 248)		
		*n*	%	*n*	%	*n*	%	
Anthracycline-based neoadjuvant treatment	Yes	595	86.2	387	87.6	208	83.9	0.218
	No	95	13.8	55	12.4	40	16.1	
Platinum/taxane-based neoadjuvant treatment	Yes	657	95.2	426	96.4	231	93.1	0.085
	No	33	4.8	16	3.6	17	6.9	
Immunotherapy Received	No	675	97.8	431	97.5	244	98.4	0.590
	Yes	15	2.2	11	2.5	4	1.6	
Completed neoadjuvant treatment	No	45	6.6	23	5.3	22	8.9	0.076
	Yes	639	93.4	415	94.7	224	91.1	
Type of surgery	Total mastectomy	405	58.7	236	53.4	169	68.1	<0.001 *
	Breast-conserving surgery	285	41.3	206	46.6	79	31.9	
Axillary surgery type	Axillary dissection	370	53.6	198	44.8	172	69.4	<0.001 *
	Sentinel lymph node dissection	320	46.4	244	55.2	76	30.6	
Pathological response	Non-pCR	460	66.7	233	52.7	227	91.5	<0.001 *
	pCR	230	33.3	209	47.3	21	8.5	
Pathological T stage	pCR	262	38.0	222	50.2	40	16.1	<0.001 *
	Non-pCR	428	62.0	220	49.8	208	83.9	
Pathological N stage	ypN0	446	64.6	368	83.3	78	31.5	<0.001 *
	ypN1–3	244	35.4	74	16.7	170	68.5	
Adjuvant radiotherapy	No	94	13.6	66	14.9	28	11.3	0.204
	Yes	596	86.4	376	85.1	220	88.7	
Adjuvant capecitabine	No	372	53.9	272	61.5	100	40.3	<0.001 *
	Yes	318	46.1	170	38.5	148	59.7	
Recurrence	No	484	70.1	352	79.6	132	53.2	<0.001 *
	Yes	206	29.9	90	20.4	116	46.8	
Mortality	Alive	506	73.3	370	83.7	136	54.8	<0.001 *
	Dead	184	26.7	72	16.3	112	45.2	
		Mean ± SD Median (Min.–Max.)		Mean ± SD Median (Min.–Max.)		Mean ± SD Median (Min.–Max.)		*p*
Follow-up duration (years)		7.47 ± 2.73 6.84 (2.77–104.43)		7.20 ± 2.55 6.64 (2.77–19.17)		7.95 ± 2.97 7.31 (3.60–20.08)		<0.001 *
OS (yeras)		4.32 ± 2.33 3.89 (0.69–13.73)		4.44 ± 2.29 4.00 (0.69–13.73)		4.11 ± 2.38 3.66 (0.75–13.00)		0.039 *
DFS (years)		3.87 ± 2.29 3.45 (0.60–13.00)		4.11 ± 2.16 3.62 (0.69–12.79)		3.44 ± 2.47 2.69 (0.60–13.00)		0.001 *

Abbreviations: pCR: pathological complete response; ypN: pathological lymph node stage after neoadjuvant therapy; OS: overall survival; DFS: disease-free survival. Mean: Mean; SD: Standard deviation; Med.: Median; Min.: Minimum; Max.: Maximum. The Chi-square (χ^2^) test was used for comparisons of categorical variables, while the independent-samples *t*-test was used for comparisons of continuous variables. Statistical significance was accepted as * *p* < 0.05. Continuous variables are presented as mean ± standard deviation (SD) and median (min–max).

**Table 3:** Comparison of Overall Survival (OS), Disease-Free Survival (DFS), and 5-Year Survival Rates According to CPS-EG Score.

Variables	Group	Median (Years) ± SE	%95 CI	5-Year OS/DFS (%)	*p*
OS	CPS-EG Score ≤ 3	10.95 ± 0.37	10.22–11.68	83.7	<0.001 *
	CPS-EG Score > 3	6.07 ± 0.94	4.23–7.90	54.1	
DFS	CPS-EG Score ≤ 3	12.70 ± 4.06	4.74–20.65	76.4	<0.001 *
	CPS-EG Score > 3	6.34 ± 1.63	3.16–9.53	51.9	
OS (non-pCR patients)	CPS-EG Score ≤ 3	9.14 ± 0.94	7.31–10.98	76.3	<0.001 *
	CPS-EG Score > 3	5.33 ± 0.77	3.82–6.83	50.5	
DFS (non-pCR patients)	CPS-EG Score ≤ 3	12.70 ± 4.08	4.70–20.69	66.5	<0.001 *
	CPS-EG Score > 3	3.93 ± 1.39	1.21–6.66	51.9	

Abbreviations: pCR, pathological complete response; OS, overall survival; DFS, disease-free survival. Data are presented as median survival ± standard error (SE) and 95% confidence interval (CI) according to Kaplan–Meier analysis. Differences between groups were evaluated using the log-rank test. * *p* < 0.05 was considered statistically significant.

**Table 4:** Results of Univariate and Multivariate Cox Regression Analyses for Overall Survival (OS).

Variables	Reference	Univariate HR (95% CI)	*p*	Multivariate HR (95% CI)	*p*
Age (≥50 vs. <50)	<50	0.77 (0.57–1.04)	0.084	–	–
Menopausal status (Post vs. Pre)	Premenopausal	0.74 (0.55–0.99)	0.044 *	0.56 (0.40–0.80)	0.001 *
ECOG (≥1 vs. 0)	ECOG 0	1.29 (0.94–1.76)	0.120	1.46 (1.01–2.10)	0.042 *
Comorbidity (Yes vs. No)	No	0.88 (0.65–1.21)	0.432	–	–
Tumor location	Lateral		0.042 *		
Medial vs. Lateral	Lateral	1.49 (1.06–2.09)	0.021 *	–	–
Other vs. Lateral	Lateral	1.39 (0.94–2.04)	0.098	–	–
Histologic subtype (Non-IDC vs. IDC)	IDC	1.20 (0.87–1.65)	0.270	–	–
Clinical T stage (T3–4 vs. T1–2)	T1–2	2.08 (1.55–2.80)	<0.001 *	–	–
Clinical N stage (N+ vs. N0)	N0	3.92 (2.12–7.23)	<0.001 *	–	–
HER2 status (Low vs. Zero)	HER2-zero	1.46 (1.08–1.96)	0.013 *	1.40 (1.02–1.94)	0.039 *
LVI (Present vs. Absent)	Absent	1.98 (1.47–2.67)	<0.001 *	1.33 (0.96–1.85)	0.091
PNI (Present vs. Absent)	Absent	1.78 (1.20–2.64)	0.004 *	–	–
Tumor grade (III vs. I–II)	I–II	1.19 (0.89–1.59)	0.255	–	–
Ki-67 (≥70 vs. <70)	<70	0.80 (0.59–1.09)	0.158	0.98 (0.70–1.37)	0.889
Platinum/taxane-based treatment (Yes vs. No)	No	1.88 (1.04–3.37)	0.036 *	–	–
Axillary surgery type (ALND vs. SLNB)	SLNB	0.54 (0.40–0.74)	<0.001 *	–	–
Pathological response (pCR vs. non-pCR)	non-pCR	0.20 (0.12–0.32)	<0.001 *	0.39 (0.23–0.67)	<0.001 *
ypT1-4 vs. ypT0)	ypT0	3.60 (2.43–5.33)	<0.001 *	–	**–**
ypN1-3 vs. ypN0)	ypN0	3.67 (2.71–4.96)	<0.001 *	1.85 (1.25–2.73)	0.002 *
Adjuvant radiotherapy (Yes vs. No)	No	1.21 (0.77–1.91)	0.409	–	**–**
CPS-EG (>3 vs. ≤3)	≤3	3.08 (2.29–4.15)	<0.001 *	1.46 (1.08–1.98)	0.015 *

Abbreviations: ALND: axillary lymph node dissection; SLNB: sentinel lymph node biopsy; HR: Hazard ratio; CI: Confidence interval. Significant variables were included in the multivariate model. The discriminative ability of the model was found to be C-index = 0.556. * *p* < 0.05 was considered statistically significant.

**Table 5:** Results of Univariate and Multivariate Cox Regression Analyses for Disease-Free Survival (DFS).

Variables	Reference	Univariate HR (95% CI)	*p*	Multivariate HR (95% CI)	*p*
Age (≥50 vs. <50)	<50	0.72 (0.54–0.95)	0.020 *	–	–
Menopausal status (Post vs. Pre)	Premenopausal	0.71 (0.54–0.93)	0.014 *	0.67 (0.50–0.89)	0.006 *
ECOG (≥1 vs. 0)	ECOG 0	1.18 (0.87–1.60)	0.296	–	–
Comorbidity (Yes vs. No)	No	0.80 (0.59–1.07)	0.132	–	–
Tumor location	Lateral	–	0.022 *	–	**–**
Medial vs. Lateral	Lateral	1.38 (0.99–1.92)	0.054	–	–
Other vs. Lateral	Lateral	1.56 (1.10–2.22)	0.014 *	–	–
Histologic subtype (Non-IDC vs. IDC)	IDC	1.18 (0.87–1.59)	0.284	–	–
Clinical T stage (T3–4 vs. T1–2)	T1–2	1.74 (1.31–2.32)	<0.001 *	–	–
Clinical N stage (N+ vs. N0)	N0	3.28 (1.93–5.57)	<0.001 *	–	–
HER2 (Low vs. Zero)	HER2-zero	1.60 (1.22–2.12)	<0.001 *	–	–
LVI (Present vs. Absent)	Absent	1.89 (1.42–2.51)	<0.001 *	1.36 (1.00–1.83)	0.047 *
PNI (Present vs. Absent)	Absent	1.72 (1.18–2.50)	0.004 *	–	–
Tumor grade (III vs. I–II)	I–II	1.28 (0.97–1.69)	0.086	–	–
Ki-67 (≥70 vs. <70)	<70	0.85 (0.63–1.14)	0.270	–	–
Platinum/taxane-based treatment (Yes vs. No)	No	1.66 (0.94–2.91)	0.079	–	–
Axillary surgery type (ALND vs. SLNB)	SLNB	0.55 (0.41–0.73)	<0.001 *	–	–
Pathological response (pCR vs. non-pCR)	non-pCR	0.29 (0.20–0.42)	<0.001 *	0.53 (0.34–0.84)	0.007 *
ypT1-4 vs. ypT0	ypT0	2.79 (2.00–3.91)	<0.001 *	–	–
ypN1-3 vs. ypN0	ypN0	3.37 (2.55–4.45)	<0.001 *	1.85 (1.30–2.64)	<0.001 *
Adjuvant radiotherapy (Yes vs. No)	No	0.82 (0.57–1.19)	0.304	–	–
CPS-EG Score (>3 vs. ≤3)	≤3	2.83 (2.15–3.73)	<0.001 *	1.73 (1.26–2.38)	<0.001 *

Abbreviations: ALND: Axillary lymph node dissection; SLNB: Sentinel lymph node biopsy; HR: Hazard ratio; CI: Confidence interval. Significant variables were included in the multivariate model. The discriminative ability of the model was found to be C-index = 0.571. * *p* < 0.05 was considered statistically significant.

## Data Availability

The datasets generated and/or analyzed during the current study are not publicly available due to ethical and privacy restrictions but are available from the corresponding author upon reasonable request.

## Supplementary Materials

The following supporting information can be downloaded at https://www.mdpi.com/article/10.3390/cancers18142302/s1, Table S1: Missing data in key variables; Table S2: Sensitivity analyses for CPS-EG after adjusting for capecitabine; Table S3: Interaction analyses; Table S4: HER2-low stratified analyses for CPS-EG.

## Abbreviations

The following abbreviations are used in this manuscript:

TNBC	Triple-negative breast cancer
NACT	Neoadjuvant chemotherapy
CPS-EG	Clinical–Pathologic Scoring System incorporating Estrogen Receptor status and Grade
pCR	Pathological complete response
DFS	Disease-free survival
OS	Overall survival
HER2	Human epidermal growth factor receptor 2
ECOG	Eastern Cooperative Oncology Group
FISH	Fluorescence in situ hybridization
