# Supplementary material for: Prognostic Significance of the CPS-EG Score in Triple-Negative Breast Cancer Treated with Neoadjuvant Chemotherapy
Source: Cancers (Basel). 2026 Jul 17;18(14):2302. doi: 10.3390/cancers18142302 (PMC13406532; doi:10.3390/cancers18142302)
Supplement: Supplementary file 1 [file cancers-18-02302-s001.zip › cancers-4398803-supplementary.pdf]

Supplementary Table S1. Missing data in key variables

Variable	Missing n/N	Missing %
Histologic subtype	42/690	6.1%
LVI	58/690	8.4%
PNI	78/690	11.3%
Ki-67 category	28/690	4.1%
NACT completion	6/690	0.9%
BRCA status	470/690	68.1%
DFS time	1/690	0.1%

Supplementary Table S2. Sensitivity analyses for CPS-EG after adjusting for capecitabine

Outcome	Model	CPS-EG >3 HR (95% CI); p	Capecitabine HR (95% CI); p	N/events	C-index
OS	Primary multivariable + capecitabine	1.64 (1.15-2.34); p=0.006	1.12 (0.80-1.58); p=0.506	613/163	0.740
DFS	Primary multivariable + capecitabine	1.72 (1.25-2.36); p=<0.001	1.13 (0.82-1.56); p=0.438	631/193	0.721
OS	Patients not receiving capecitabine	1.67 (0.95-2.92); p=0.072	Not applicable	314/68	0.784
DFS	Patients not receiving capecitabine	2.17 (1.30-3.61); p=0.003	Not applicable	330/79	0.763

Supplementary Table S3. Interaction analyses

Outcome	Interaction term	HR (95% CI); p
OS	CPS-EG >3 x capecitabine	1.03 (0.53-2.03); p=0.921
OS	CPS-EG >3 x platinum/taxane	1.57 (0.37-6.62); p=0.541
OS	CPS-EG >3 x anthracycline	0.97 (0.38-2.47); p=0.946
OS	CPS-EG >3 x immunotherapy	Not interpretable (very sparse subgroup)
OS	CPS-EG >3 x HER2-low	1.05 (0.55-2.02); p=0.885
DFS	CPS-EG >3 x capecitabine	0.61 (0.33-1.12); p=0.108
DFS	CPS-EG >3 x platinum/taxane	1.36 (0.39-4.70); p=0.629
DFS	CPS-EG >3 x anthracycline	0.65 (0.28-1.52); p=0.324
DFS	CPS-EG >3 x immunotherapy	Not interpretable (very sparse subgroup)
DFS	CPS-EG >3 x HER2-low	1.12 (0.62-2.01); p=0.709

Supplementary Table S4. HER2-low stratified analyses for CPS-EG

Outcome	HER2 subgroup	CPS-EG >3 HR (95% CI); p	N/events	C-index
OS	zero	1.73 (1.10-2.71); p=0.017	412/99	0.728
DFS	zero	1.71 (1.11-2.63); p=0.015	419/110	0.701
OS	low	1.77 (0.98-3.19); p=0.056	201/64	0.742
DFS	low	1.81 (1.11-2.95); p=0.018	212/83	0.735
